# Herniated Bladder Diverticulum Through the Obturator Foramen Mimicking Pelvic Lymph Node on FDG-PET: A Case of Avoided Complication During CT-Guided Biopsy

**DOI:** 10.7759/cureus.97798

**Published:** 2025-11-25

**Authors:** Rahul Jain, Alec Dallas, David Mina, Vaiva Gustainyte, Elias Salloum

**Affiliations:** 1 Interventional Radiology, University of South Florida Health Morsani College of Medicine, Tampa, USA; 2 Interventional Radiology, Moffitt Cancer Center, Tampa, USA

**Keywords:** bladder diverticulum, diagnostic pitfall, fdg pet-ct, interventional radiology, lymph node biopsy, pelvic lymph node

## Abstract

Fludeoxyglucose (FDG)-positron emission tomography (PET)/computed tomography (CT) is commonly used in cancer staging, but urinary tract activity and anatomical variations can resemble lymph node disease, leading to unnecessary treatments and potentially harmful procedures. Here, we present the case of a patient referred to Interventional Radiology for a CT-guided biopsy of a suspicious pelvic lymph node with FDG uptake on outside PET/CT. The lesion was located between the pectineus and obturator muscles in the left pelvis. During needle insertion, the changing appearance of the lesion prompted the procedure to be paused for further review of available imaging and careful examination. Imaging re-evaluation revealed the target to be a small bladder diverticulum with a long, narrow neck herniating through the obturator foramen, filled with radiotracer-laden urine. The procedure was aborted, and the biopsy was deferred, avoiding potential complications such as bladder perforation or infection. This experience highlights the importance of thorough multimodal correlation and intraprocedural vigilance to identify benign mimics of nodal disease and ensure patient safety.

## Introduction

Fludeoxyglucose (FDG)-positron emission tomography (PET)/computed tomography (CT) is a key component of cancer staging, offering essential guidance for diagnosis and treatment planning [1]. Hypermetabolic foci, particularly in pelvic lymph nodes, are often believed to indicate metastatic disease [2]. However, FDG also accumulates in the urinary tract, and benign anatomical variants, such as bladder diverticula, can resemble nodal disease [3]. This pitfall becomes more pronounced when diverticula are narrow-necked, extend into unusual anatomic planes, or appear separated from the bladder dome [4]. It can also be missed if the diverticula have long, thin connecting stalks that lie between imaging slices [5]. Misinterpretation can result in unnecessary biopsy requests and potential complications, such as bladder perforation or infection [6]. Therefore, careful multimodality correlation is crucial to ensuring patient safety [7].

## Case presentation

**Figure 1:**
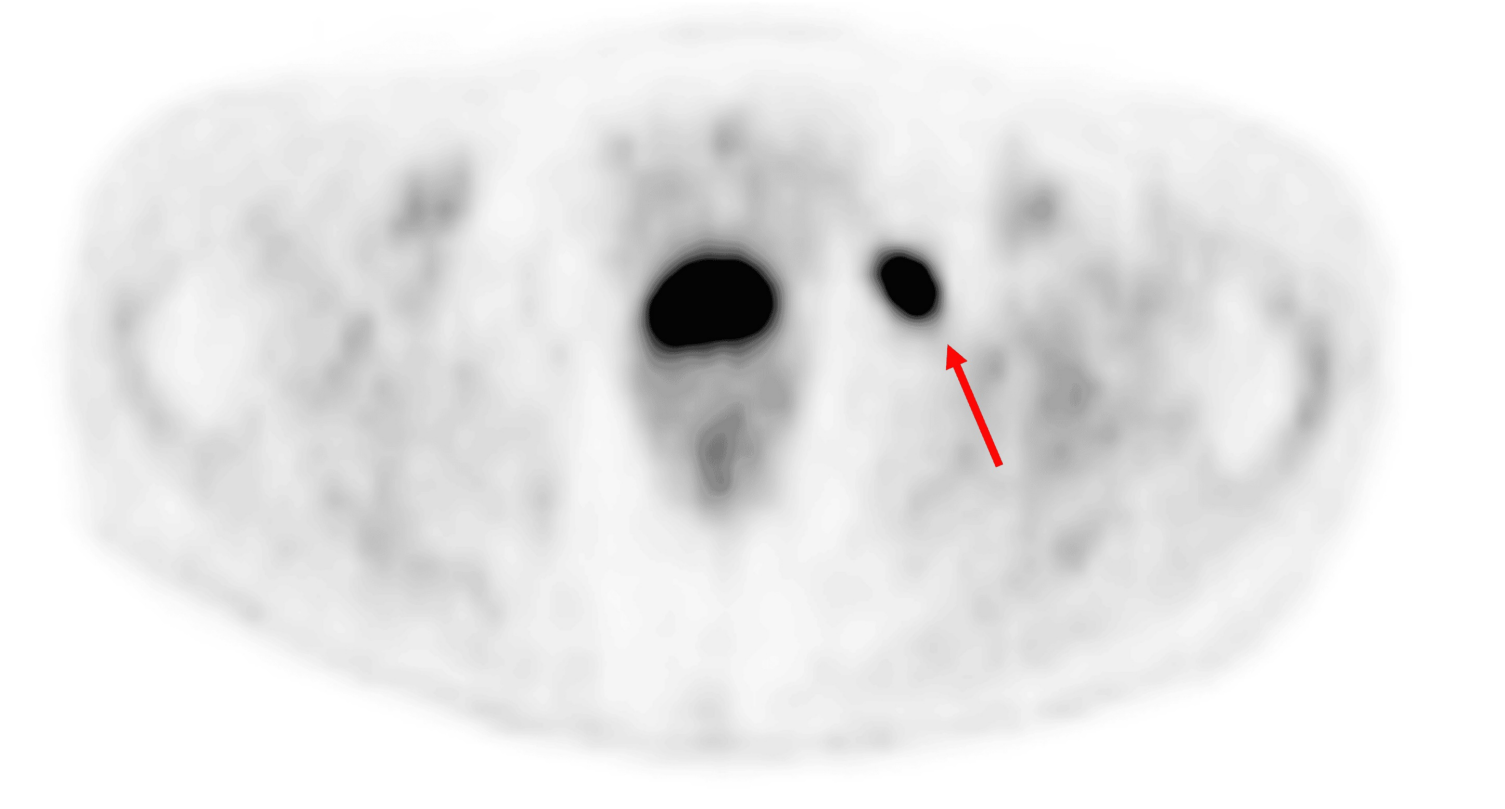
Positron emission tomography image demonstrating an fludeoxyglucose-avid area (maximum standardized uptake value of 11.6) of concern in the left pelvis. This was described as an avid “lymph node” on outside imaging and was the target requested for a computed tomography-guided biopsy.

A 78-year-old female with a history of diffuse large B-cell lymphoma undergoing treatment had received an FDG-PET/CT at an outside hospital, which reported an FDG-avid left pelvic “lymph node” (Figure 1). The lesion demonstrated focal uptake between the pectineus and obturator muscles in the left pelvis, raising concern for disease progression and triggering a biopsy request. The patient was scheduled for a CT-guided pelvic “lymph node” biopsy with Interventional Radiology.

**Figure 2:**
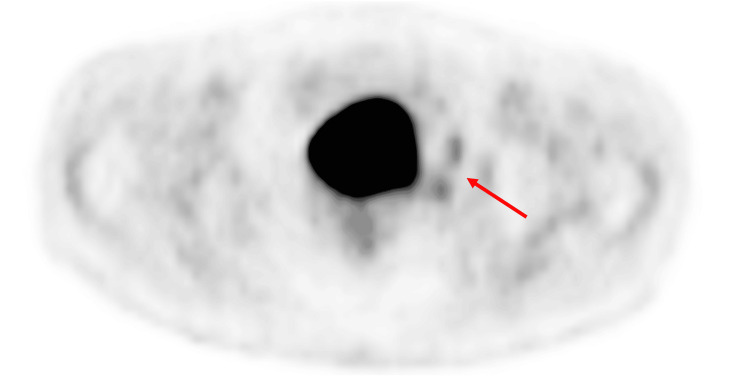
A more cranial positron emission tomography/computed tomography slice demonstrates a hint of focal uptake suspected to be the neck of the diverticulum extending from the bladder.

**Figure 3:**
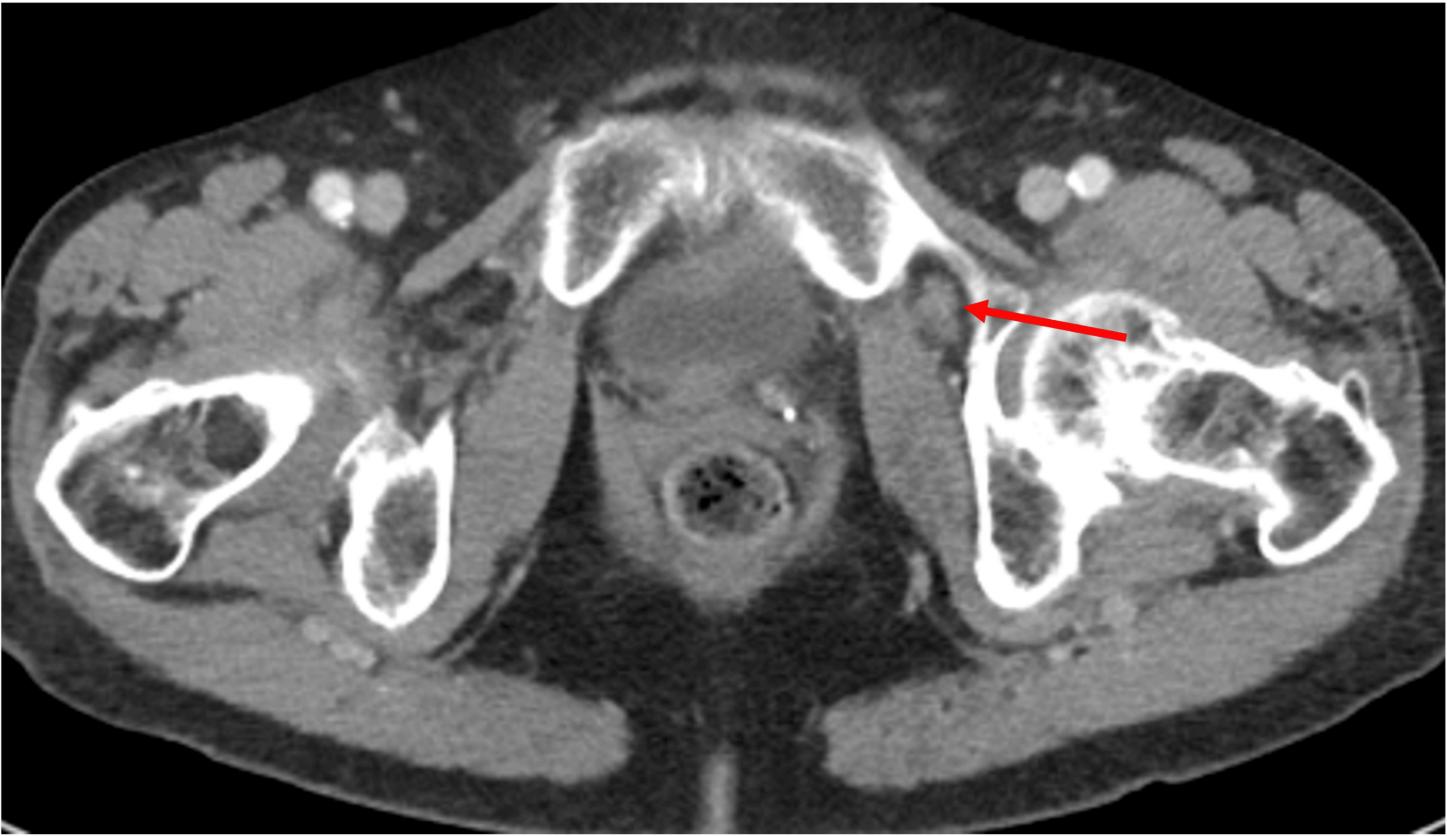
Contrast-enhanced axial computed tomography image demonstrating the decompressed bladder diverticula within the obturator foramen.

**Figure 4:**
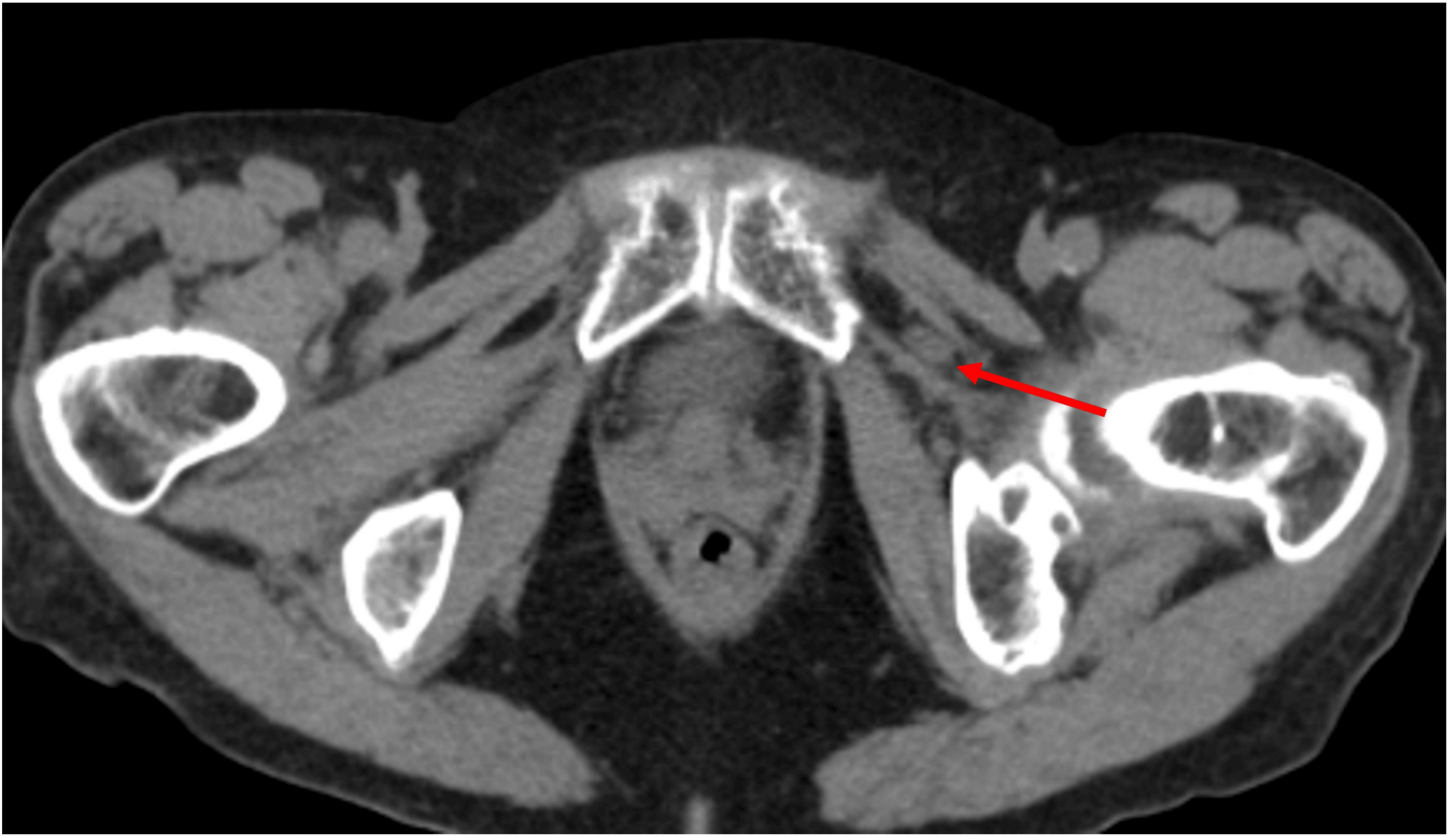
Contrast-enhanced axial computed tomography image demonstrating the decompressed bladder diverticula terminating between the pectineus and obturator muscles.

**Figure 5:**
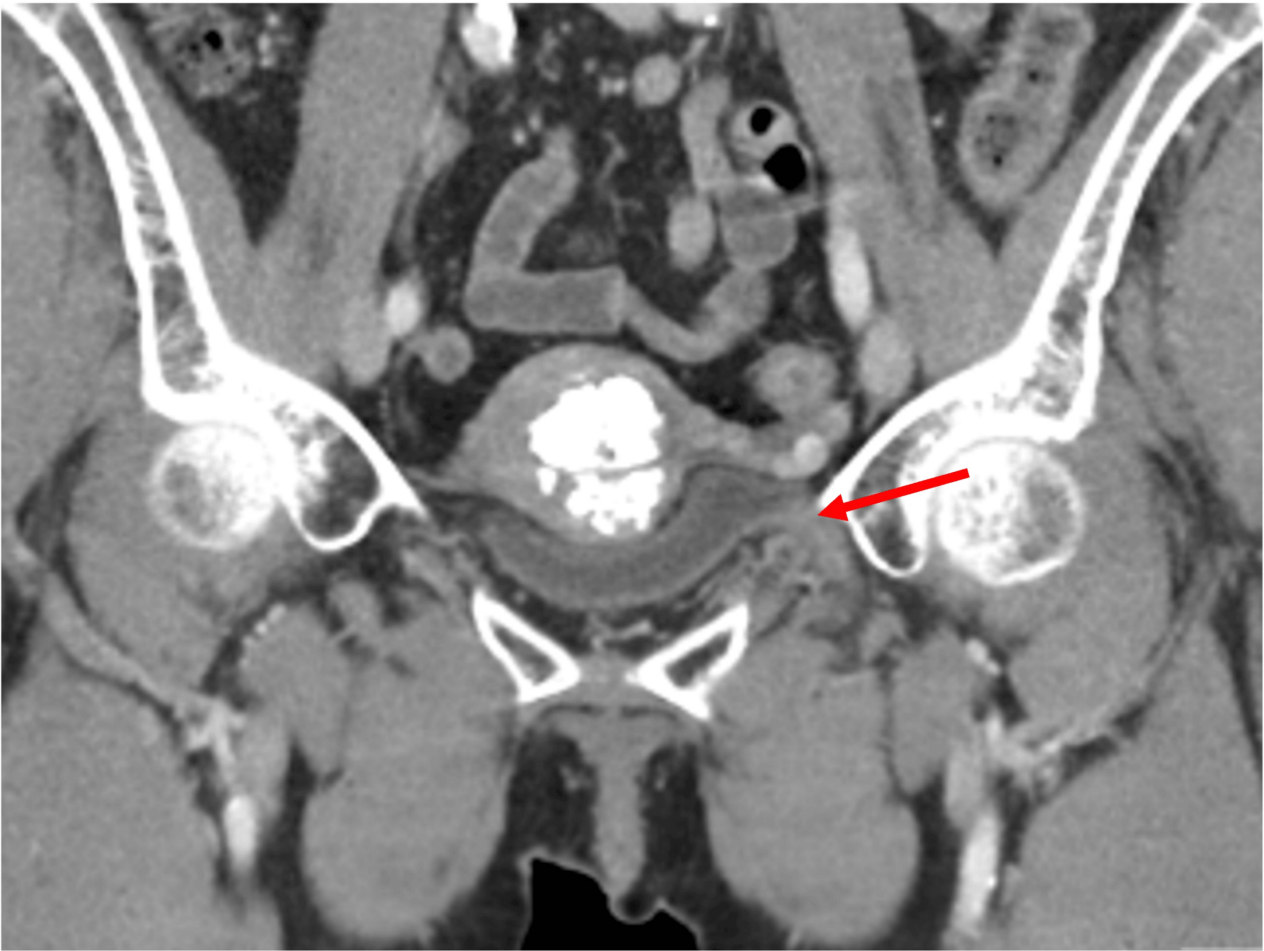
Contrast-enhanced coronal computed tomography image depicting the narrow-neck origin of a bladder diverticulum as it arises from the bladder wall and courses through the obturator foramen.

**Figure 6:**
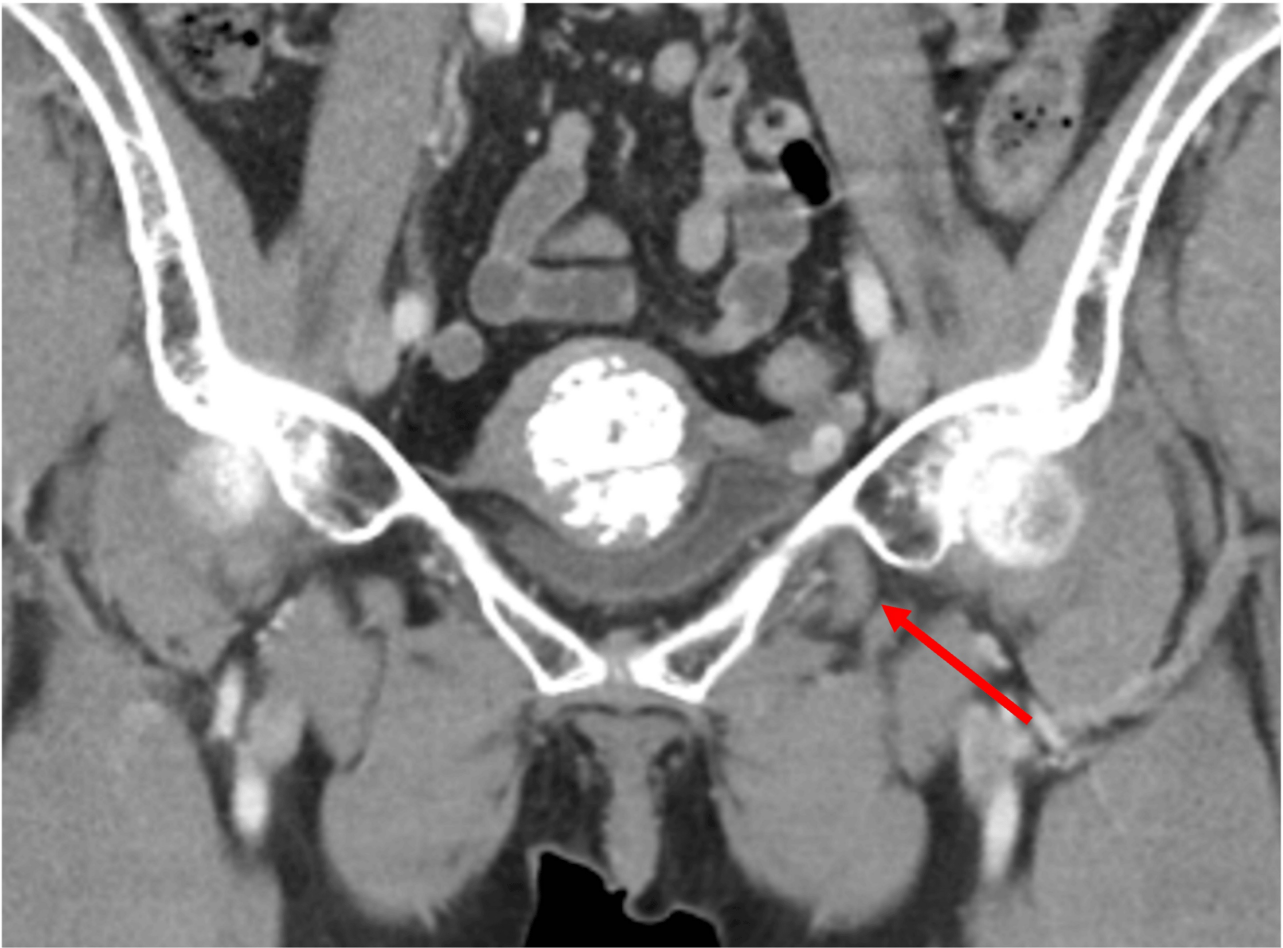
Contrast-enhanced coronal computed tomography image redemonstrating the decompressed diverticula as it passes through the obturator foramen.

**Figure 7:**
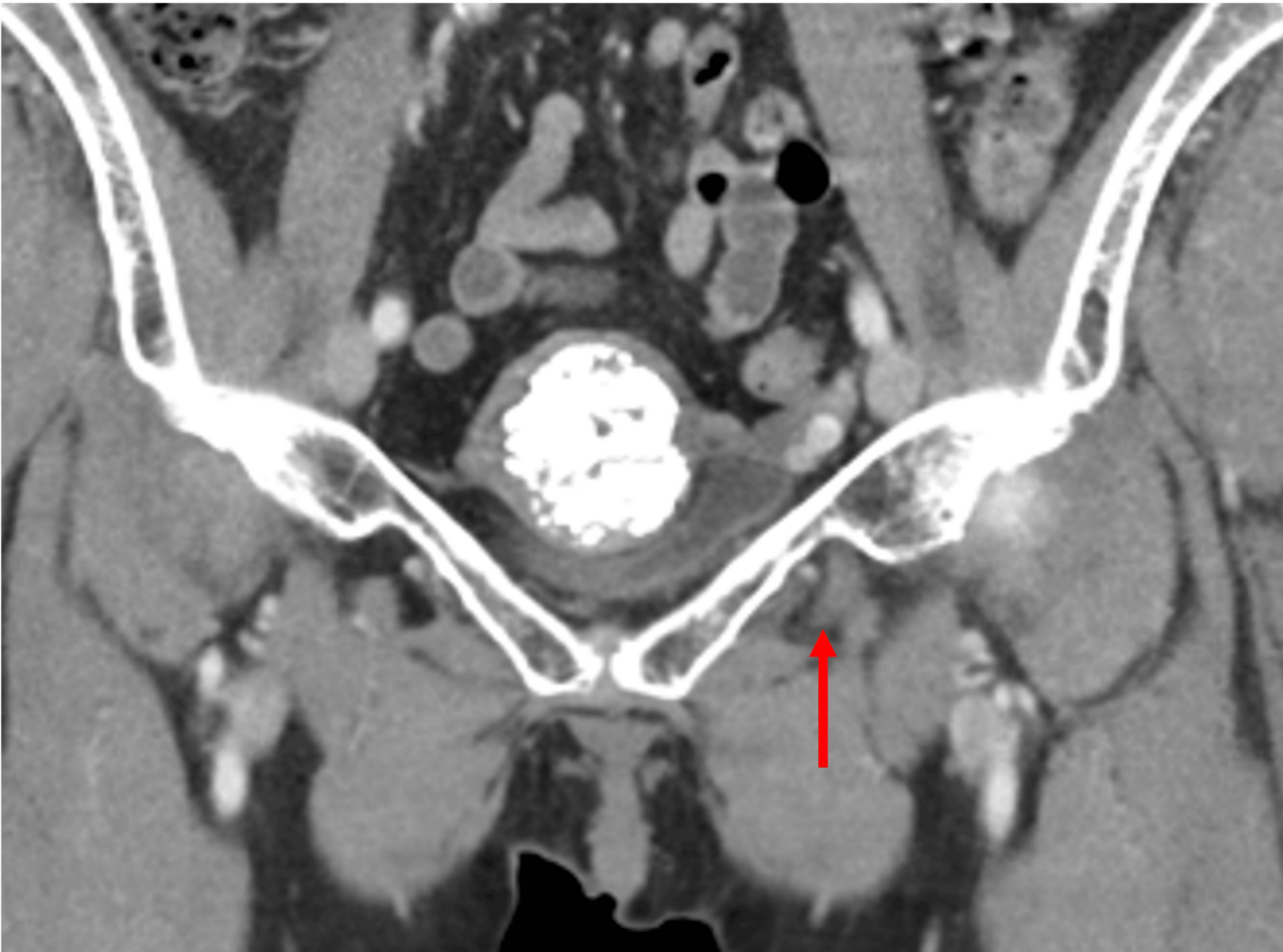
Contrast-enhanced coronal computed tomography image showing the distal extent of the decompressed bladder diverticulum as it passes through and terminates within the obturator foramen.

On the day of the procedure, a scout CT redemonstrated the left pelvic lesion. After standard sterile technique and injection of 1% lidocaine, the Interventional Radiologist began by advancing the biopsy needle under CT guidance. The lesion’s morphology, however, shifted dynamically between scans, atypical of solid nodal tissue [8]. The procedure was then paused, and the Interventional Radiologist reevaluated the outside PET/CT and compared it with the intraprocedural imaging. After closer review, the “lymph node” was, in fact, a bladder diverticulum with a long, narrow neck that herniated through the obturator foramen. The FDG uptake (maximum standardized uptake value of 11.6) on the outside PET/CT was attributed to radiotracer-containing urine within the diverticulum, rather than malignant activity [4]. Upon closer examination, a faint hint of a suspected diverticular neck can be seen on the outside PET/CT image in Figure 2. The procedure was then deferred to prevent possible complications such as iatrogenic bladder puncture or infection. Subsequent follow-up diagnostic imaging demonstrated the diverticulum extending between the pectineus and obturator muscles on contrast-enhanced axial CT images (Figures 3, 4). Contrast-enhanced coronal CT images demonstrated the diverticular origin as it bud off the bladder (Figure 5), coursed through the obturator foramen (Figure 6), and terminated in the left pelvis (Figure 7).

## Discussion

Although the principles of PET/CT are well known in the medical community, a brief discussion is helpful for context. PET/CT imaging uses an intravenously injected tracer, most often FDG, glucose tagged with radioactivity, to identify areas of tracer accumulation indicating increased metabolic activity.

False positives can result from various sources such as normal physiological uptake, inflammatory processes, or benign anatomical variants [4,9]. Active inflammation or infection may show tracer accumulation that resembles malignancy [10]. Normal uptake in metabolically active organs such as the brain, heart, and kidneys can also pose interpretive problems, especially when lesions are nearby [11]. Other error sources include artifacts caused by metallic implants, dental fillings, contrast media, or treatment-related inflammation, such as that from chemotherapy regimens containing rituximab [12]. Meanwhile, false negatives can occur in tumors with low metabolic activity (e.g., low-grade lymphomas or adenomas) that do not take up enough tracer for detection [13]. Small lesions, under 1 cm, may also be below the scanner’s resolution capacity [14]. Additionally, lesions near active tissues may be hidden or hard to distinguish, leading to more false-negative results [15]. Bladder diverticula and mucosal outpouching of the detrusor wall are examples of such anatomical variants. They may result from outlet obstruction, infection, or congenital weakness [16]. On imaging, they can resemble cystic or nodal structures, especially when filled with radiotracer-laden urine. The risk of misdiagnosis rises when the diverticulum extends beyond the bladder contour into atypical anatomic planes such as the obturator foramen. They may also be difficult to identify if the diverticulum is connected to the bladder by a long, thin stalk, which can lie between imaging slices [17].

Several reports in the literature describe bladder diverticula masquerading as pelvic masses or lymph nodes on PET/CT, highlighting the diagnostic challenge [18]. Failure to recognize this mimic may result in unnecessary treatments, biopsies, or surgery, exposing patients to avoidable complications such as bladder perforation, hemorrhage, or infection [19]. In this case, real-time procedural vigilance and correlating PET/CT findings with anatomical knowledge enabled the Interventional Radiologist to identify the lesion as a bladder diverticulum, preventing iatrogenic injury.

This case underscores several important learning points. First, a thorough review of all available imaging before the procedure is essential, especially when PET/CT findings are unusual in appearance or location. Second, reassessment during the procedure is crucial when lesion behavior diverges from expectations, such as changes in size or shape over serial images. Lastly, close collaboration between interpreting radiologists and proceduralists enhances patient safety by ensuring that potential issues are identified and properly managed. However, this report’s limitations include its single-patient design, which limits broad application. Additionally, factors such as bladder filling and radiotracer excretion can influence imaging appearance, emphasizing the importance of careful interpretation of similar findings.

Additionally, we want to highlight the suboptimal quality of external imaging, which can greatly impair diagnostic interpretation and clinical decision-making [20]. In this case, the external PET scans shown in Figure 1 and Figure 2 were of low quality and, therefore, difficult to interpret, creating a false impression of a separate lesion and resulting in an unnecessary biopsy request. This underscores the importance of image quality in accurate diagnosis, as low-resolution or poorly acquired scans can lead to misinterpretation, diagnostic uncertainty, and potentially avoidable procedures.

## Conclusions

A bladder diverticulum herniating through the obturator foramen can closely resemble pelvic nodal disease on FDG-PET/CT. Correlating PET findings with cross-sectional imaging and maintaining procedural vigilance are essential to prevent misdiagnosis and unnecessary interventions or treatments. Interventional radiologists should stay aware of this potential pitfall and remain cautious when lesion features do not match typical nodal characteristics or locations. Ultimately, careful review and intraprocedural flexibility are key to protecting patients and achieving optimal outcomes.
